# A dual drug regimen synergistically blocks human parainfluenza virus infection

**DOI:** 10.1038/srep24138

**Published:** 2016-04-07

**Authors:** Benjamin Bailly, Larissa Dirr, Ibrahim M. El-Deeb, Ralf Altmeyer, Patrice Guillon, Mark von Itzstein

**Affiliations:** 1 Institute for Glycomics, Gold Coast Campus, Griffith University, Gold Coast, 4222, Australia; 2 Unit of anti-infective research, Institut Pasteur of Shanghai – Chinese Academy of Sciences, Shanghai, 200031, P.R. China; 3 Shandong University-Helmholtz Institute of Biotechnology, Qingdao, 266101, P.R. China

## Abstract

Human parainfluenza type-3 virus (hPIV-3) is one of the principal aetiological agents of acute respiratory illness in infants worldwide and also shows high disease severity in the elderly and immunocompromised, but neither therapies nor vaccines are available to treat or prevent infection, respectively. Using a multidisciplinary approach we report herein that the approved drug suramin acts as a non-competitive *in vitro* inhibitor of the hPIV-3 haemagglutinin-neuraminidase (HN). Furthermore, the drug inhibits viral replication in mammalian epithelial cells with an IC_50_ of 30 μM, when applied post-adsorption. Significantly, we show in cell-based drug-combination studies using virus infection blockade assays, that suramin acts synergistically with the anti-influenza virus drug zanamivir. Our data suggests that lower concentrations of both drugs can be used to yield high levels of inhibition. Finally, using NMR spectroscopy and *in silico* docking simulations we confirmed that suramin binds HN simultaneously with zanamivir. This binding event occurs most likely in the vicinity of the protein primary binding site, resulting in an enhancement of the inhibitory potential of the *N*-acetylneuraminic acid-based inhibitor. This study offers a potentially exciting avenue for the treatment of parainfluenza infection by a combinatorial repurposing approach of well-established approved drugs.

Human parainfluenza viruses (hPIVs) are the second most prevalent cause of acute respiratory tract infection in infants, after human respiratory syncytial virus. Among parainfluenza viruses, hPIV-3 is the principal causative agent of disease in infants^1^,^2^,^3^. Apart from being mostly infectious towards children under 2 years of age, they also cause severe respiratory symptoms in the elderly, immunocompromised and transplant patients^4^,^5^,^6^. Despite the clinical significance of hPIVs, neither drugs nor vaccines are available to either treat or prevent infection.

hPIV-3 has two surface envelope glycoproteins, a haemagglutinin-neuraminidase (HN) and a fusion protein (F). HN plays three roles in the life-cycle of the virus namely, (1) attachment of the virus to host cell sialylglycoconjugate receptors that contain α2-3 or α2-6-linked terminal sialic acids (in humans, *N*-acetylneuraminic acid, Neu5Ac) residues^7^,^8^; (2) cleavage of sialic acid entities to promote the release of virus progeny from the cell^1^,^9^ and (3) finally stabilisation of F and initiation of fusion^10^,^11^,^12^,^13^,^14^,^15^. The fact that HN is involved in so many critical steps makes it an ideal target for anti-parainfluenza virus drug design^16^,^17^,^18^,^19^.

Most of the compounds that have been investigated as anti-hPIV-3 agents are competitive inhibitors of HN. They are primarily *N*-acylneuraminic acid derivatives based on Neu5Ac2en (DANA, *N*-acetyl-2,3-didehydro-2-deoxy-neuraminic acid), one of the first inhibitors of a broad range of neuraminidases (sialidases)^20^. The influenza virus neuraminidase inhibitor zanamivir^21^,^22^ or derivatives of the hPIV inhibitor BCX-2798 have been the most widely studied, with the latter compounds displaying some prophylactic efficacy in a mouse model of hPIV-3 infection^17^,^23^,^24^ (**1–4**, Fig. 1).

In an effort to contribute to the discovery of potent inhibitors of hPIV-3 infection, we have recently published a series of studies focused on the hPIV-3 HN protein. We demonstrated, using Molecular Dynamics simulations, that the protein possesses a flexible loop in the vicinity of the active site around Asp216. The cavity formed by an open 216 loop can accommodate more of the receptor than just the terminal Neu5Ac moiety of a sialylglycoconjugate that may provide broader receptor specificity and contribute to host cell tropism^25^. Moreover, we have synthesised and evaluated several potent sialic acid-based inhibitors, including a difluoro-derivative of BCX-2798 that was shown to covalently bind the key catalytic residue Tyr530 in the HN active^26^, as well as a bulky C4-substituted phenyltriazole derivative of BCX-2798 (**5**, Fig. 1) that was shown to be nicely accommodated into the protein’s active site and to lock open the 216-loop upon engagement^19^.

In the present study we look into another strategy to block HN-mediated hPIV-3 infection, by investigating approved drugs, not related to *N*-acylneuraminic acids, that may inhibit hPIV-3 HN haemagglutination and neuraminidase functions. Such drugs could provide an alternative approach for hPIV-3 infection blockade and have an advantage for potential repositioning. Through this approach, we have successfully identified suramin (**7**), a trypanocidal drug commonly prescribed for the treatment of sleeping sickness in Africa^27^,^28^,^29^, as a non-competitive inhibitor of HN receptor binding and neuraminidase functions. Moreover, we have demonstrated that the drug has significant *in vitro* antiviral potency and acts synergistically when combined with competitive inhibitors of HN. Our study shows that compounds other than *N*-acylneuraminic acid derivatives can indeed inhibit neuraminidases by binding, in the case of the hPIV-3 HN, in a region of importance in close proximity to its active site.

## Results

### Enzyme-based semi-high throughput screening of hPIV-3 neuraminidase activity inhibitors

**Figure 1:**
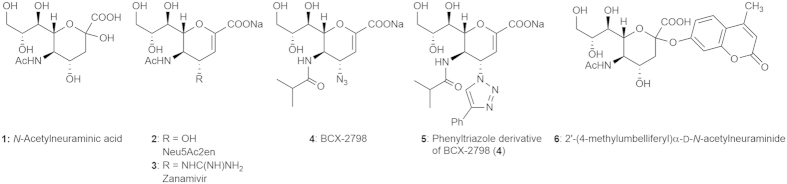
Structures of *N*-acylneuraminic acid-based inhibitors of hPIV-3 HN. Chemical structures of *N*-acetylneuraminic acid (Neu5Ac, **1**), Neu5Ac2en (**2**), zanamivir (**3**), BCX-2798 (**4**), the phenyltriazole derivative of BCX-2798 (**5**) and 2′-(4-methylumbelliferyl) α-D-*N*-acetylneuraminide (MUN, **6**). Ph = phenyl, Ac = acetyl.

In our desire to discover novel, repurposable drugs of hPIV-3, we screened a library of 1280 USA and Internationally approved drugs in a neuraminidase activity inhibition assay using the neuraminidase substrate 2′-(4-methylumbelliferyl) α-D-*N*-acetylneuraminide (MUN, **6**, Fig. 1). The drugs, stored in DMSO, were tested at a final concentration of 100 μM. This concentration allowed dissolution of drug in an aqueous buffer, with an acceptable DMSO concentration (1% final), and resulted in a less than 20% loss of fluorescence signal (data not shown). The quality of the assay was determined by Z-factor calculation^30^. Throughout the screening we obtained an average Z-factor = 0.74 ± 0.19, indicating that the assay was reliable and within a good dynamic range.

**Figure 2:**
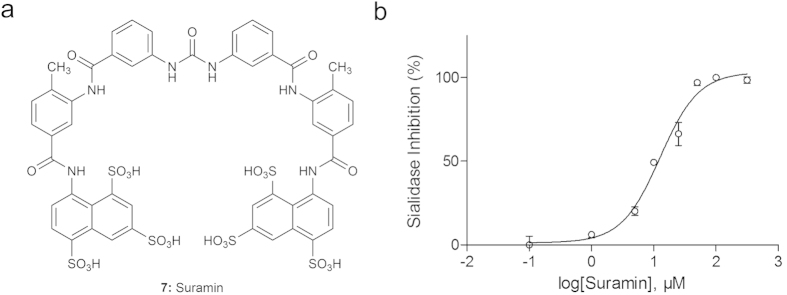
Enzymatic inhibition of HN by suramin (7). (**a**) Chemical structure of suramin (**7**). (**b**) Dose-response of suramin (**7**) against hPIV-3 neuraminidase activity. Data points are the mean of duplicate values and are representative of a least 2 independent experiments. The error bars represent the standard error of the mean (SEM).

In primary screening, a threshold of 80% reduction of neuraminidase activity was chosen to discriminate compounds with an IC_50_ above 100 μM. Out of 1280 drugs, three inhibitors of the hPIV-3 neuraminidase activity were identified and confirmed in a secondary screening (data not shown): suramin (**7**, Fig. 2a), Evans blue and erythrosine sodium, with a neuraminidase inhibition of 94 ± 0.2%, 79.4 ± 1.8% and 92.8 ± 0.9%, respectively. As Evans blue and erythrosine sodium are dyes, their quenching effect on fluorescence was evaluated by adding the compounds at the end of the enzymatic reaction. No decrease in fluorescence was observed, showing no quenching effect of the dyes. However, due to the nature of the compounds, they were not further evaluated as inhibitors of hPIV-3 in this study.

### Suramin (7) inhibits the hPIV-3 HN haemagglutinin and neuraminidase activities in a dose-dependent manner

The potency of suramin (**7**) as an inhibitor of hPIV-3 HN neuraminidase activity was further characterised by dose-response HN neuraminidase activity inhibition (NI) experiments. The NI assay was carried out following the conditions of the primary screening, using serial dilutions of drug. Suramin (**7**) was found to inhibit HN neuraminidase in a dose-dependent manner, with an IC_50_ of 13.5 μM (Fig. 2b). As a comparison, compound **5** had a reported IC_50_ of 1.5 μM^19^ (Table 1).

As the HN neuraminidase active site is also an *N*-acylneuraminic acid-containing glycan binding site, we tested whether the drug could block the haemagglutinin activity of the protein. In this haemagglutination inhibition assay (HI), we challenged the capacity of HN to agglutinate guinea pig red blood cells (gpRBC) in the presence of suramin (**7**). We qualitatively observed the agglutination of gpRBC after 1 h 30 min of incubation with the virus and the drug at 4 °C. The dose required for suramin (**7**) to inhibit 50% of the HN haemagglutinin activity was ~200 μM, compared with 2.7 μM for compound **5 **19 (Table 1).

### Suramin (7) inhibits the hPIV-3 HN neuraminidase activity via a non-competitive mechanism

**Figure 3:**
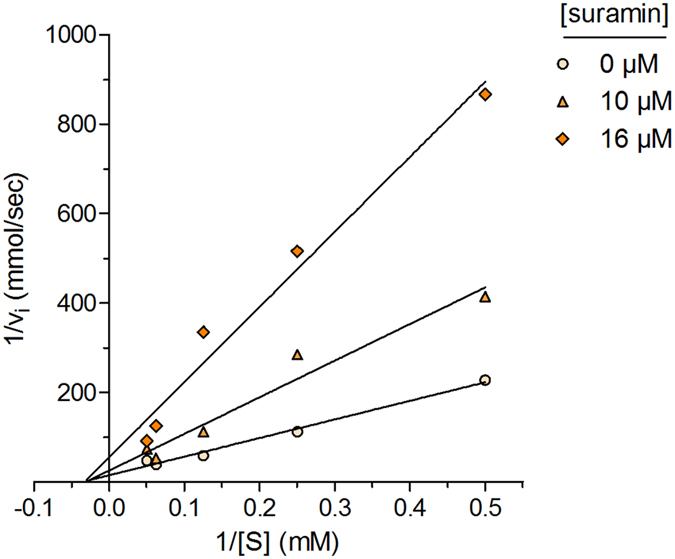
Determination of the inhibition mode of suramin (7) by enzyme kinetics. The initial velocities *v*_*i*_ of the HN neuraminidase activity were determined at several concentrations of the substrate MUN (2, 4, 8, 16, 20 mM, **6**) for each concentration of suramin [suramin]. The Lineweaver-Burke graph was created by plotting duplicate values of 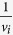 as a function of 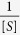 according to Equation (1), and is representative of 3 independent experiments. The straight lines are linear regressions calculated for each concentration of inhibitor.

Although suramin (**7**) has been shown to inhibit some neuraminidases, its mechanism of action remains unknown^31^,^32^,^33^. To understand the mode of inhibition of the drug on HN, we conducted an enzyme kinetics analysis of the neuraminidase activity in presence of suramin (**7**) and the substrate MUN (**6**). In the setup of the neuraminidase activity inhibition experiment, we evaluated the initial velocity *v*_*i*_ of the neuraminidase at several substrate concentrations ([S]). At each of these [S], we challenged the enzyme with a dilution range of inhibitor concentrations ([I]). Linear regressions of data points from Lineweaver-Burk plots using Equation (1) were undertaken for each concentration of suramin (**7**, Fig. 3). They all converge and cross the X-axis at a single value. The Michaelis-Menten constant (K_M_) value, which approximately corresponds to the HN affinity for the substrate, was calculated from this point of convergence and found to be of 30.3 ± 1.02 mM. The inhibition constant (K_i_) of suramin (**7**) was equal to 5.06 ± 0.62 μM. In contrast, the enzyme’s maximum velocity (V_max_) was 0.068 mmol/sec and was reduced when suramin (**7**) was present in the reaction with apparent maximum velocities (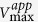) values of 0.04 and 0.019 mmol/sec at drug concentrations of 10 and 16 μM, respectively. These data strongly suggest that **7** acts on the hPIV-3 HN via a non-competitive mechanism. Therefore, it does not bind directly to the protein primary binding site and does not compete with *N*-acetylneuraminic acid-containing conjugates or small molecules.

### Suramin (7) has *in vitro* antiviral activity

**Figure 4:**
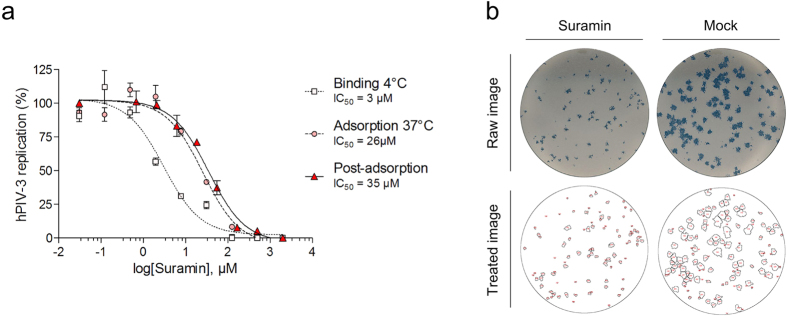
*In vitro* antiviral effect of suramin (7) on hPIV-3-infected LLC-MK2 cells. Dose-dependent inhibition of hPIV-3 infection by suramin (**7**) at different stages of infection (**a**). The antiviral potencies of the drugs were evaluated by focus reduction assay, and the drugs were added either during virus binding (4 °C for 1 h), at adsorption stage (37 °C for 1 h), or post-adsorption (37 °C for 72 h). Foci numbers (virus binding and adsorption at 37 °C) or foci size (post-adsorption) were used to determine viral replication. Immunostaining was carried out after 72 h of incubation. (**b**) Post-adsorption effect of suramin (**7**) on reduction of foci size at 30 μM as compared to an untreated control (mock). Top: scan of a focus reduction assay from a 24-well plate immunostained 72 h post-infection. Bottom: image of the same well after image conversion to a binary image and particle detection for automated foci counting and size measurements using Fiji. Each detected focus is outlined in black and numbered in red.

We evaluated the dose-dependent antiviral potency of suramin (**7**) on hPIV-3-infected LLC-MK2 cells by immunostained focus reduction assay. We evaluated the drug at binding stage stage at 4 °C and adsorption stage at 37 °C for an hour to evaluate the effect on virus binding (4 °C) as well as early events of infection including fusion (37 °C). In another experiment the drug was added post-virus adsorption at 37 °C to evaluate post-internalisation effects. Interestingly, suramin (**7**) had the most potent antiviral effect during virus binding at 4 °C with an IC_50_ value of 3.1 μM, showing that the drug efficiently blocked hPIV-3 HN receptor binding site and prevented entry (Fig. 4a). Suramin (**7**) also inhibited infection during adsorption at 37 °C (binding and fusion events) but with a lesser efficacy (IC_50_ = 26 μM), and post-adsorption with an IC_50_ value of 35 μM. As shown on Fig. 4b, the reduction of the average size of foci by suramin (**7**) could be accurately measured using automated high-resolution image treatments. While the number of foci remained constant, their size could be reduced down to the size of single infected cells.

### Suramin (7) acts in synergy with competitive HN inhibitors to block *in vitro* infection

**Figure 5:**
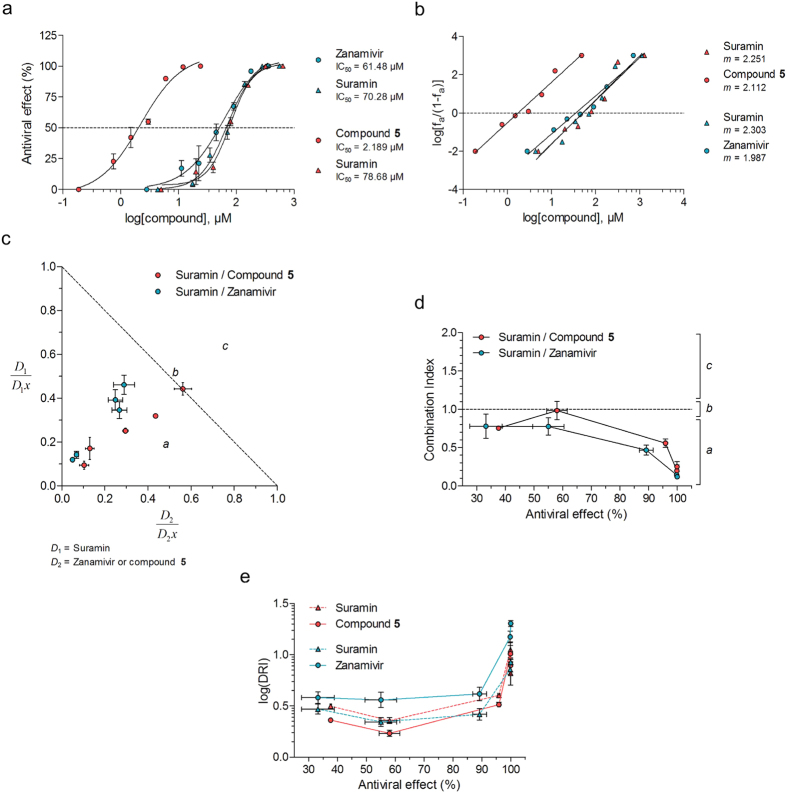
Evaluation of the synergism of suramin (7) in combination with competitive *in vitro* inhibitors of HN. Data sets in red and blue correspond to suramin (**7**)—compound **5** or suramin (**7**)—zanamivir (**3**) combinations, respectively. (**a**) Dose-response curves of each individual compound. Suramin (**7**) was evaluated twice, for each of the combinations with suramin and compound **5**. The antiviral effect was determined by measurement of foci size. (**b**) Median-effect representation of the dose-response curves for each individual compound, using Equation (4). *m* is the linear regression slope, *f*_*a*_ is the “fraction affected”, or (% effect) ÷100. (**c**) Normalised isobologram that represents, for each combination, the normalised dose of each compound individually required to reach the observed effect in combination (Equation (3)). *D*_1_ is the dose of a compound 1 in combination required to achieve *x*% of inhibition, while *D*_*x*1_ is the dose of a compound alone required to achieve *x*% of inhibition. Data points in zone *a, b* and *c* correspond to combinations with synergistic, additive or antagonistic effects, respectively. (**d**) CI-effect plot representing the combination index CI, calculated using Equation (2), of each combination as a function of their associated antiviral effect. The zones *a, b* and *c* are the same as the ones described in (**c**). (**e**) log(DRI)-effect plot representing the drug reduction index (DRI) of compounds as a function of their antiviral effect in combination. The DRI is calculated for each drug in each combination according to Equation (5), and represents the dilution factor required for a drug to reach the same level of inhibition individually compared with it when in combination. All combinations were tested in quadruplicate, post-adsorption, by a focus reduction assay. The results are representative of 3 independent experiments.

Since suramin (**7**) is a non-competitive inhibitor of HN showing antiviral potency *in vitro*, it may be expected to have a synergistic effect when combined with a competitive inhibitor, as both compounds would not bind the same region of the protein. Furthermore, as suramin (**7**) is an approved drug, we tested this hypothesis with the approved anti-influenza drug and competitive hPIV-3 HN inhibitor, zanamivir (**3**) to see if lower concentrations of both drugs could be used to block infection *in vitro*. We also evaluated combinations with compound **5**, a recently designed hPIV-3 HN inhibitor, to observe if a synergy could also be observed with a better competitive inhibitor. We used the method of Chou and Talalay to design the experiments and assess the various combinations^34^,^35^,^36^. The drugs were tested at constant ratios of equipotent concentrations, ranging from 0.25 to 4-fold of their respective IC_50_ value that was determined for each experiment (Fig. 5a, Table 2). To mimic treatment rather than prophylaxis, we applied drugs post-virus adsorption.

In this particular assay, suramin (**7**) had an average IC_50_ value of 74.5 μM, compared with 61.5 μM for zanamivir (**3**) and 2.2 μM for compound **5**. The slope m was also determined from a linear regression of median-effect plots (Equation (4)), as they reflect the sigmoidicity of the dose-response curves and was used for the subsequent calculation of normalised concentrations and drug reduction indices (Fig. 5b). The antiviral effects of individual compounds and compounds used in combination experiments are provided in Table 2. The normalised isobologram is a graphical way of visualising synergistic combinations with regard to concentrations. Since *D*_1_ is the concentration of compound 1 responsible for an effect *x* in combination, and *D*_1_*x* the concentration of compound 1 responsible for an effect *x* on its own, a normalised concentration 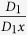, calculated using Equation (3), tends to zero as lower concentrations of compounds in combination are required to reach an effect *x*. As shown in Fig. 5c, virtually all data points are located in region *a* where combinations have a synergistic effect, suggesting that both zanamivir (**3**) and compound **5** act in synergy with suramin (**7**) to efficiently block hPIV-3 infection. The CI-effect plot also allows the visualisation of combination effects, based on the combination index CI calculated using Equation (2). It represents the CI value as a function of the associated antiviral effect for each combination, and a CI value <1, >1 and = 1 is representative of synergistic, antagonistic and additive effects, respectively (Fig. 5d). Similarly to Fig. 5c, all combination points to the exception of one were inferior to 1, indicating a synergistic effect between suramin (**7**) and zanamivir (**3**) or compound **5**.

The fact that the compounds show synergism means that their concentration in combination yields an effect that is stronger than individual compounds at a similar or higher concentration. This property of synergistic inhibitors can be evaluated via a calculation of the drug reduction index (DRI, Equation (5)), for each molecule from each combination, and is represented with the log(DRI)-effect plot (Fig. 5e). In the case of suramin (**7**)—zanamivir (**3**) or suramin (**7**)—compound **5** combinations to inhibit *in vitro* hPIV-3 infection, the stronger the effect, the higher the DRI. Although this result is as expected, it does not translate to high synergism as the DRI is calculated for individual drugs at a given effect in combination. Based on the DRI-effect plot, 100% inhibition of infection in combination is reached when the concentration of a compound can be reduced more than 10-fold as compared with the concentration needed to reach the same effect, individually. In addition, as shown in Table 2, only 2-times the IC_50_ value of suramin (**7**) and zanamivir (**3**), and 2-times the IC_50_ value of suramin (**7**) and compound **5** used in combination are sufficient to inhibit 100% of *in vitro* hPIV-3 infection, compared with 4-times the IC_50_ value for any drug used individually.

### Suramin (7) binds to a site distinct from hPIV-3 HN primary binding site

Because suramin (**7**) and competitive inhibitors of HN work in cooperativity to block *in vitro* infection, and because HN possesses a unique, dual-function active site, we hypothesised that suramin (**7**) may bind in close proximity to the active site. To verify this hypothesis, and to confirm the non-competitive inhibition of suramin (**7**), we followed another approach using a competition-Saturation Transfer Difference (STD)-NMR experiment in presence of the approved drug zanamivir (**3**). STD NMR spectroscopy is a dynamic technique that enables the determination of how ligands engage proteins in solution^19^ and if they compete with known inhibitors^19^,^37^. We analysed the change in binding of zanamivir (**3**) to purified hPIV-3 particles upon addition of suramin (**7**), as well as the change in binding of suramin (**7**) upon addition of zanamivir (**3**).

**Figure 6:**
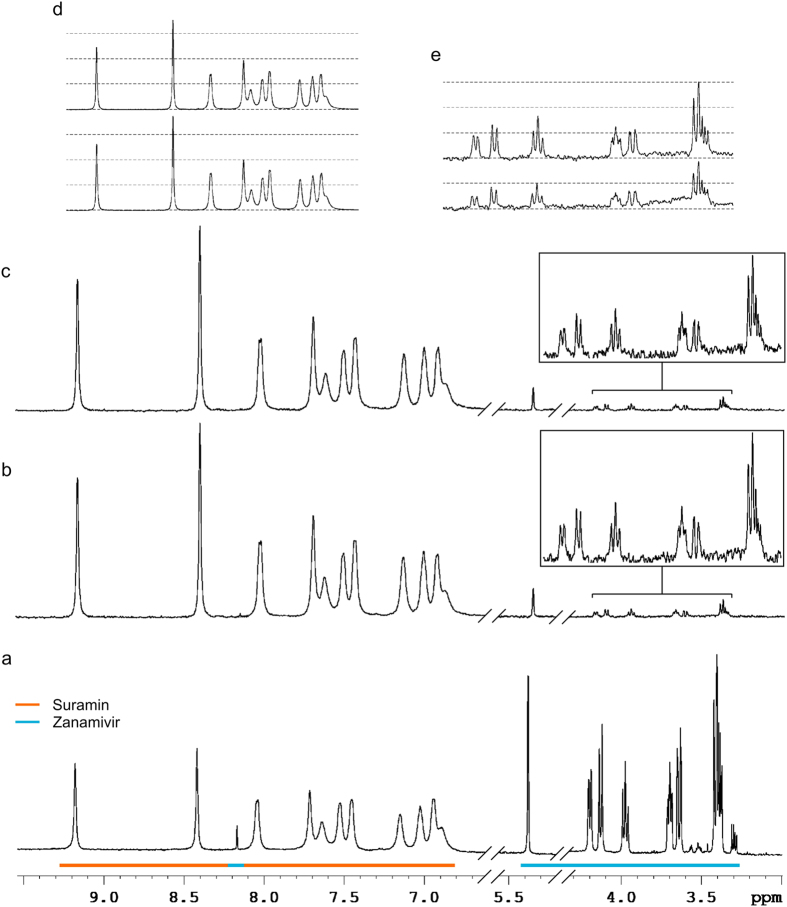
Competition STD-NMR of suramin (7) and zanamivir (3) in presence of purified hPIV-3. (**a**) ^1^H-NMR spectrum of suramin (**7**) and zanamivir (**3**). (**b**,**c**) Competition STD-NMR spectra of suramin (**7**) and zanamivir (**3**) in presence of virus where zanamivir (**3**) was added before (**b**) or after (**c**) suramin (**7**). Absolute intensities of STD NMR signals (**b**,**c**) are comparable. (**d**) STD-NMR spectra of suramin (**7**) alone in presence of virus (bottom), and after addition (top) of zanamivir (**3**). (**e**) STD-NMR spectra of zanamivir (**3**) alone in presence of virus (bottom), and after (top) addition of suramin (**7**). Drugs in combination were tested at an equimolar ratio.

As expected, their ^1^H NMR signals were equivalent in intensities (Fig. 6a) and STD-NMR signals of both drugs were obtained, thus confirming their binding to HN (Fig. 6b,c). The STD-NMR signals of suramin (**7**) were higher than those of zanamivir (**3**), mainly due to the perfect symmetry of the molecule and to the consequent amplification of the signal of each proton.

The sequential order in which the drugs were added to the biological sample did not influence the final STD-NMR spectra of the drug combinations (Fig. 6b **versus c**). The STD-NMR intensities of suramin (**7**) remained unchanged upon addition of zanamivir (**3**, Fig. 6d). The STD-NMR intensities of zanamivir (**3**), however, were increased by ~30% upon addition of suramin (**7**, Fig. 6e). Similar intensities were obtained when suramin (**7**) was added before zanamivir (**3**), which suggests that suramin (**7**) binding to HN modifies the architecture of the primary binding site, thus influencing the zanamivir (**3**) binding event.

**Figure 7:**
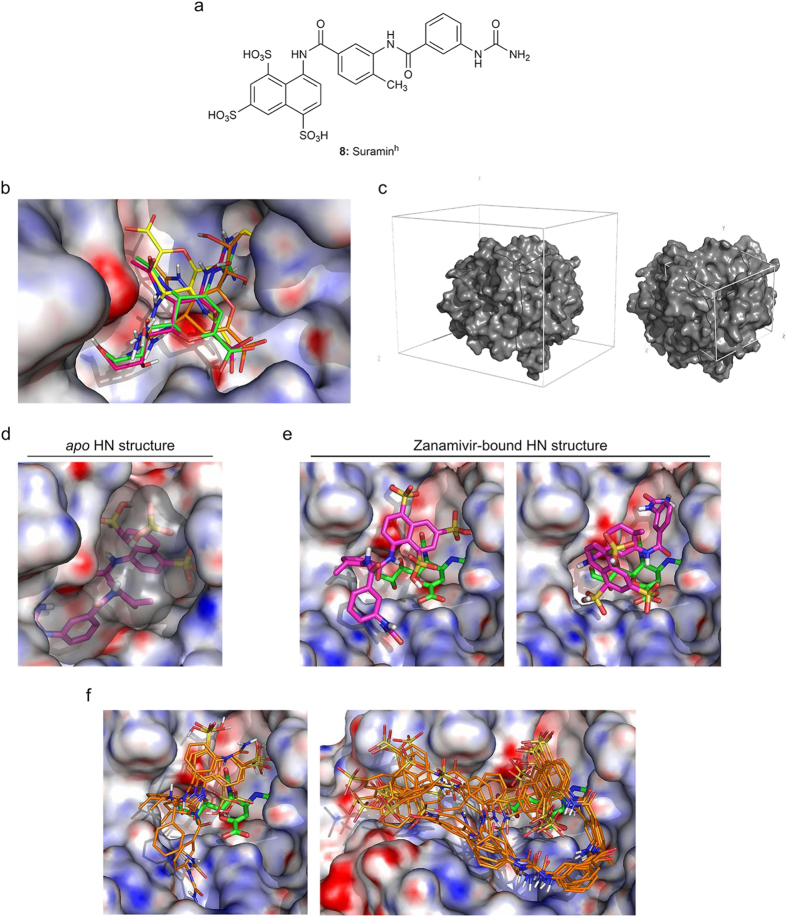
Probing of suramin^h^ (8) in the hPIV-3 HN binding site. (**a**) Chemical structure of Suramin^h^ (**8**), used for blind docking simulations. (**b**) Solvent-accessible surface representation of HN active site (1V3E), with zanamivir (**3**) docked. Zanamivir (**3**) from the structure 1V3E is represented in green, the 3 best conformations of zanamivir (**3**) from docking simulations are represented in magenta, orange and yellow. (**c**) Suramin^h^ (**8**) binding site probing strategy. A total of 36 overlapping grids (search spaces) were designed to cover the entire surface of an HN monomer (represented in grey), and simulations were run on each of them. Left: volume of the 36 grids combined. Right: volume of a single grid centred on the HN active site. (**d**) Solvent-accessible surface representation of HN with the best conformation of **8** docked to the active site of the *apo*-form (1V3B, suramin^h^ (**8**): in magenta, grey surface). (**e**) Solvent-accessible surface representations of HN with the 2 lowest energy conformations of **8** resulting from 2 overlapping search spaces positioned over the active site of zanamivir (**3**)-bound HN (1V3E; zanamivir (**3**): in green; suramin^h^ (**8**): in magenta). (**f**) Solvent-accessible surface representation of HN with a dominant cluster of suramin^h^ (**8**, left) and suramin (**7**, right) docked over the active site of zanamivir (**3**)-bound HN (1V3E; zanamivir (**3**): in green; suramin^h^ (**8**) and suramin (**7**): in orange. The HN surface in (**b**,**d**–**f**) is coloured from red (−20 kT/e) to blue (20 kT/e) according to the electrostatic potential (APBS).

To establish a putative binding site for suramin (**7**) on HN, we performed some initial *in silico* docking simulations using available hPIV-3 HN structures (1V3B, 1V3E)^38^. In the first instance, to simplify our simulations we took advantage of the symmetrical nature of suramin (**7**) and reduced the drug’s structure to leave a terminal R-NHCONH_2_ moiety with R being the polycyclic half of the molecule (**8**, suramin^h^, Fig. 7a). As a positive control for docking simulations, zanamivir (**3**) from the available structure 1V3E was re-docked into the HN active site. As shown in Fig. 7b, the conformations of the most favourable binding energies overlapped with the crystal structure of zanamivir (**3**). The simulated yellow and orange zanamivir structures were in opposite orientations when compared to zanamivir (**3**, green) bound in the original crystal structure, however the simulated magenta zanamivir (**3**) structure was positioned very close to the original zanamivir (**3**, green)-bound structure with an RMSD = 0.46 Å (RMSD: root mean square deviation).

We then designed a blind-docking simulation experiment to probe the potential binding site of the suramin^h^ (**8**) molecule on hPIV-3 HN using Autodock Vina on 36 overlapping search spaces covering an entire monomer (Fig. 7c). A total of 10 suramin^h^ (**8**) binding conformations were generated for each of the 36 search spaces. In the *apo*-structure, the lowest energy structure of suramin^h^ (**8**) was positioned in the HN active site (Fig. 7d). The experimentally-determined kinetics previously led us to conclude that **7** is a non-competitive inhibitor and consequently does not compete with substrate or relevant active site-binding inhibitors. Interestingly, in the zanamivir (**3**)-bound structure the 2 lowest energy complexes with **8** were identified near the HN active site where **8** covered zanamivir (**3**) (Fig. 7e). A refined docking of **8** on the same structure confirmed this prediction, as shown by the 3-conformation-containing cluster represented in Fig. 7f, **left**. These results suggest, using suramin^h^ (**8**) as a probe, that suramin (**7**) is most likely to bind in the vicinity of the HN active site. To confirm our prediction of the orientation of suramin (**7**) to the HN active site region, dockings were performed on the zanamivir (**3**)-bound structure using the full suramin structure **7**. Fig. 7f, **right**, shows a dominant cluster of 8 conformations of **7**. In this cluster, the naphthalene trisulfonate groups engage basic residues within the HN active site, while the rest of the molecule fills the space in front of the HN binding site. Further refined simulations of **7** and **8** on the *apo* structure failed to yield significant clustering of the naphthalene trisulfonate moieties, although binding was restricted to the active site region.

## Discussion

Our approach in this study was to discover novel scaffolds of hPIV-3 HN neuraminidase activity inhibitors that could be repositionable drugs and work either synergistically with other inhibitors (or drugs), or act as a tool to better understand the mechanism by which the protein’s functions are regulated. Following a target-based drug discovery strategy, we identified the approved drug suramin (**7**) as a novel inhibitor of hPIV-3 HN. We found that it acts in synergy with competitive inhibitors of HN to block hPIV-3 *in vitro* infection and thus is a non-competitive inhibitor for the enzyme.

We determined the anti-hPIV-3 neuraminidase activity of suramin (**7**) by enzyme-based semi-high throughput screening of approved drugs on the neuraminidase function of HN. This is, to our knowledge, the first such study conducted on human parainfluenza virus neuraminidase. The drug, not related to the sialic acid family, was found to have activity both on the neuraminidase and the haemagglutinin functions of HN.

The kinetic analysis we conducted on the hPIV-3 sialidase with suramin (**7**) showed that the drug did not compete with the substrate. While the apparent maximum velocity of the enzyme changed significantly in presence of drug, its constant of affinity for the substrate remained unaffected. The binding of suramin (**7**) and the MUN (**6**) substrate to HN are therefore unaffected by one another, meaning that they bind to distinct sites on the protein.

Given that the HN protein possesses a single primary binding domain, suramin (**7**) would inhibit both entry and release *in vitro* if it bound closely to the sialic acid recognition site. We found suramin (**7**) to be the most potent at binding stage *in vitro*, although it is also active post-adsorption. This indicates that suramin (**7**) prevents the binding of HN to sialic acid-containing receptors, as well as their cleavage. The lack of molecular and cell dynamics in a 4 °C environment could be responsible for a longer-lasting binding event between suramin (**7**) and the virus and cells. Most importantly, unlike at 37 °C, at 4 °C the hPIV-3 fusion protein would not be engaged upon binding of the virus^12^,^39^, preventing spontaneous fusion events even in the presence of an entry inhibitor.

The *N*-acetylneuraminic acid-based drug zanamivir (**3**) was shown to have *in vitro* activity in several studies against hPIV-3, although its potency is in the high micromolar range^15^,^21^,^40^. This is also the case for suramin (**7**), but we have shown that the two drugs can act in synergy to inhibit hPIV-3 infection *in vitro*. As a result, we showed that the dose of both drugs can be significantly reduced to reach high levels of inhibition, as compared to the individual dose that would otherwise be needed to reach a similar effect. In this study we also demonstrated that this feature of suramin (**7**) can be applied to better competitive inhibitors of hPIV-3 HN, such as compound **5**. Similar levels of synergy as with zanamivir (**3**) can be achieved in combination with compound **5**, but as it is more potent than zanamivir (**3**) even lower concentrations of drugs are needed. This data also confirms the non-competitive inhibition nature of suramin (**7**). Indeed, if suramin (**7**) bound to the active site, either suramin (**7**) or the competitive inhibitor would occupy the identical binding pocket and no synergism would be observed. However, suramin (**7**) must bind in proximity to the HN binding site in order to block the protein’s functions.

To further investigate this property of suramin (**7**), we conducted competition STD-NMR experiments with suramin (**7**) and zanamivir (**3**) using purified virus particles. Suramin (**7**) appeared to bind more strongly than zanamivir (**3**) on HN, as determined by the intensities of STD-NMR signals. Suramin (**7**) and zanamivir (**3**) were both found to bind HN, whether they were in combination or not, confirming the non-competitivity of suramin (**7**) towards HN. The STD-NMR signals of zanamivir (**3**) however were enhanced by about 30% upon addition of an equimolar ratio of suramin (**7**). Similar STD-NMR signals of zanamivir (**3**) were found when suramin was added first. To-date, only a closed-216 loop structure of hPIV-3 HN has been observed in complex with zanamivir (**3**)^25^,^38^. Since we observe an increase in STD-NMR signals for zanamivir (**3**) in the presence of suramin (**7**) and hPIV-3 HN, it suggests that a faster exchange of zanamivir (**3**) from bound- to free-form occurs^41^. A possible explanation of this outcome is that suramin (**7**) induces the 216 loop to open enabling this faster exchange.

Although suramin (**7**) is a non-competitive inhibitor of HN, our results indicate that suramin (**7**) binds most likely in the vicinity of the active site. The extensive *in silico* analysis that we conducted in the present study substantiate this hypothesis. We probed the binding site of a truncated variant of suramin, suramin^h^ (**8**), by blind docking simulations on 36 overlapping search spaces covering an entire HN monomer. In both the *apo*-form and the zanamivir (**3**)-bound forms of HN, the dockings of lowest energies were located around the active site. In a cluster of low energy structures, suramin^h^ (**8**) was shown to bind in the vicinity of the zanamivir (**3**)-bound active site. We have also shown that a full-suramin (**7**) molecule orients towards the HN active site in a zanamivir (**3**)-bound structure, as supported by a dominant conformational cluster. This suramin (**3**) binding mode extends out of the binding pocket of the protein and may alter the zanamivir binding site as well as prevent binding of longer sialylglycoconjugates. Together, these results confirm what was observed in STD-NMR experiments and *in vitro* combination assays, and suggest that both drugs act in synergy to block hPIV-3 infection.

This study presents an *in vitro* proof of concept for combinatorial drug repurposing, as both suramin (**7**) and zanamivir (**3**) are approved drugs. Furthermore, we show that such a method has the potential to be applied using suramin (**7**) in conjunction with more specific hPIV-3 HN inhibitors. Suramin (**7**), originally a trypanocidal drug, has been found to be a promising clinical candidate for the treatment of enterovirus 71 infection *in vitro* and *in vivo*^42^, showing that drug repurposing could be used for the treatment of hPIV-3 infection.

## Materials and Methods

### Cells and viruses

LLC-MK2 cells were provided by Institut Pasteur Shanghai’s Pathogen Diagnosis Centre and maintained at 37 °C; 5% CO_2_ in DMEM (Invitrogen, Carlsbad, CA) supplemented with 10% FBS and penicillin/streptomycin. The human parainfluenza type-3 virus (strain C-243) was obtained from the American Type Culture Collection (Manassas, VA) and propagated in LLC-MK2 cells in un-supplemented DMEM. Virus stocks were prepared by infecting cells at a low multiplicity of infection (MOI) of 0.1. After 3 days of incubation, the virus-containing culture supernatant was clarified by centrifugation at 2000 × *g* for 15 min at 4 °C, aliquoted and stored at −80 °C for subsequent titration by focus forming assay.

### Compounds

Compound **5** and zanamivir (**3**) were synthesised in the Institute for Glycomics, as previously described^19^,^43^. Neu5Ac2en (DANA, *N*-acetyl-2,3-didehydro-2-deoxyneuraminic acid, **2**) was purchased from J & K Chemical (Shanghai, China), and the neuraminidase substrate MUN (2′-(4-methylumbelliferyl) α-D-*N*-acetylneuraminic acid, **6**) was purchased from Sigma Aldrich (St. Louis, MO).

### PEG-precipitation of hPIV-3 particles

For neuraminidase activity inhibition assays (NI) and STD-NMR experiments, hPIV-3 prepared by propagation in LLC-MK2 cells at a MOI of 1 were precipitated by treating clarified virus-containing supernatant of infection with a 5X solution of sterile 40%PEG-6000. Tubes were kept at 4 °C for 2 h under gentle agitation, centrifuged for 15 min at 3000 × *g* at 4 °C and the supernatants were discarded. The pellets were soaked at 4 °C overnight with 1/100^th^ of the initial volume of GNTE buffer (200 mM glycine, 200 mM NaCl, 20 mM Tris-HCl, 2 mM EDTA, pH 7.5). They were resuspended on the next day by gentle up-and-down pipetting. The precipitated virus was pooled and kept at 4 °C for later use in neuraminidase assay.

For STD-NMR experiments, the hPIV-3 particles were purified on a 30–60% non-linear sucrose gradient in GNTE buffer^44^, as previously described^19^. The PEG-precipitated virus was firstly resuspended in GNTE buffer using a dounce homogeniser, and loaded onto the gradient. The particles were purified by centrifugation at 100,000 × g for 2 h 30 without break at 4 °C, using a SW 32.1 Ti Rotor (Beckman Coulter, Brea, CA).

### Enzymatic screening of hPIV-3 neuraminidase activity inhibitors

The USA-approved and International-approved drug libraries (1280 compounds) were purchased from MicroSource Discovery System, CT. The drugs were diluted in reaction buffer prior to testing in NI assay in duplicates, at a concentration of 100 μM. The compound Neu5Ac2en (DANA, *N*-acetyl-2,3-didehydro-2-deoxyneuraminic acid, **2**) was used as a positive control of inhibition at a final concentration of 10 mM (2 mM IC_50_ for hPIV-3’s HN^16^). A threshold of 80% of neuraminidase inhibition at 100 μM was chosen to discriminate hit compounds. The potency (IC_50_) of the hits was further determined by dose-response experiments.

### Neuraminidase activity inhibition (NI) assay

The anti-neuraminidase activity of compounds was evaluated by NI assay. They were performed as described previously^19^, using a modified protocol from Potier *et al*.^45^, Suzuki *et al*.^46^ and Holzer *et al*.^47^. PEG-precipitated virus (concentration yielding at least 5 times the signal of background) and compounds were diluted in GNTE buffer and reaction buffer (NaOAc 50 mM, CaCl_2_ 5 mM, pH 4.6). Into the wells of black 96-well plates was introduced 7 μL of reaction buffer, 1 μL of compound, and 1 μL of virus. The plates were pre-incubated for 30 min at 37 °C before 1 μL of the fluorogenic neuraminidase substrate MUN (2′-(4-methylumbelliferyl) α-D-*N*-acetylneuraminic acid, **6**) was added to the mixture at a final concentration of 2 mM. The plates were incubated at 37 °C for 30 min at 1000 rpm. The reaction was stopped in each well by addition of 150 μL of carbonated buffer (pH 10.8). The plates were read at an excitation wavelength of 335 nm and an emission wavelength of 460 nm in a Varioskan Flash Multimode Reader (Thermo Scientific). The Relative Fluorescence Units (RFU) values for each measurement were normalised to the blank (reaction stopped immediately after addition of substrate) and expressed as percentage of inhibition of hPIV-3 HN neuraminidase activity. The IC_50_ for NI assay has been defined as the average inhibitory concentration of a compound leading to a 50% reduction of hPIV-3 HN neuraminidase activity.

### Kinetic analysis

The inhibition mechanism of hit compounds was determined by enzyme kinetics experiments on the hPIV-3 HN protein using a modified protocol of Ryu *et al*.^48^, Nguyen *et al*.^49^ and Mishin *et al*.^50^. The neuraminidase activity of HN was measured every 2 min over a period of 20 min, at 5 concentrations of MUN (**6**) [S]: 2, 4, 8, 10 and 20 mM, and 3 concentrations of inhibitor [I]: 0, 10 and 16 μM. The initial velocity *v*_*i*_ of the enzyme for each [S]—[I] combination was determined automatically by the software provided with the Varioskan Flash Multimode Reader. A Lineweaver-Burke plot was represented for all [I], by plotting


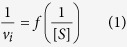


and applying a linear regression using the software GraphPad Prism version 5 (GraphPad Software, La Jolla California USA). The point of convergence of the 4 linear regressions helped determine the nature of the compound inhibition (competitive, non-competitive, un-competitive, or mixed-inhibition), and allowed the calculation of the Michaelis-Menten constant K_M_ of the enzyme for the substrate, as the intercept when Y = 0 equals 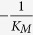. The maximum velocity *V*_max_ of the enzyme without inhibitor as well as the apparent maximum velocities 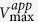 at each [I] were derived from the slopes ∆, as 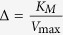. Finally, the inhibition constant K_i_ of the inhibitor for the enzyme was calculated for each [I].

### Haemagglutination inhibition assays

Guinea pig red blood cells (gpRBC) were used for the haemagglutination inhibition (HI) assay. The HN inhibitors were assessed in duplicate as reported previously^19^. Compounds were diluted in PBS as a 4X solution for each concentration tested (25 μL/well, 1X final). Each dilution was mixed with 4 haemagglutination units (HAU) of purified hPIV-3 (25 μL/well, 1 HAU final) and incubated for 20 min at room temperature. One haemagglutination unit corresponds to the lowest final concentration of virus that results in a complete agglutination of gpRBC at 4 °C. The plate was transferred on ice and an equivalent volume of ice-cold 1% gpRBC in PBS (50 μL/well) was added to each well. The plate was incubated for 1 h 30 min at 4 °C before reading haemagglutination inhibition. The IC_50_ for HI assay was defined as the compound concentration that results in a similar agglutination as the one observed in a control well containing only 0.5 HAU of hPIV-3 and gpRBC.

### Cell-based assays

The *in vitro*, dose-dependent potency of compounds was determined by immuno-stained focus reduction assay, using confluent cells in 96 or 48-well plates infected with 20–100 focus forming units (ffu) of hPIV-3 per well. To test inhibition of virus binding or adsorption (binding and fusion), compounds and virus in un-supplemented DMEM were applied to cells for 1 h at 4 °C or 37 °C, respectively. The inoculum was removed, the cells washed with PBS and overlaid with un-supplemented DMEM containing 0.75% carboxymethyl cellulose (CMC). The cells were further incubated for 72 h at 37 °C, 5% CO_2_. To assess inhibition of virus propagation, compounds were applied after 1 h of virus adsorption at 37 °C, for 72 h at 37 °C 5% CO_2_ in DMEM containing 0.75% CMC. The immuno-staining was performed by fixing cells for 20 min with 4% PFA in PBS at room temperature, followed by a 30 min incubation at 37 °C with a primary anti-hPIV-3 HN monoclonal antibody (clone M02122321, mouse IgG – Fitzgerald, Acton, MA) diluted 1:2000 in PBS-5% skim milk, and a 30 min incubation at 37 °C with a 1:6000 dilution of secondary HRP-conjugated polyclonal antibody (goat anti-mouse IgG (H + L) – Bethyl, Montgomery, TX) in PBS-5% skim milk. Virus-infected cells were stained by addition of TrueBlue Peroxidase Substrate (KPL, Gaithersburg, MD) until appearance of dark blue foci. The concentration of compound resulting in 50% inhibition (IC_50_) of virus binding (4 °C) or adsorption (37 °C) was determined by foci counting, while the IC_50_ for virus replication (post-adsorption) was determined by measurement of foci size. IC_50_ values were determined using non-linear regression analysis with the software GraphPad Prism (GraphPad Software, La Jolla California USA).

Foci counts and size measurements were done using the software Fiji^51^. Briefly, the backgrounds of 1200 dpi colour images from scanned infection plates were subtracted using a rolling ball radius setting of 10 pixels with the sliding paraboloid option. Colour channels were split and red channels (channels containing the darkest foci) smoothened before applying the threshold function to convert to binary images and the watershed function to split merged foci. The number and average size of foci in each well were determined using the particles analyser.

The toxicity of compounds towards LLC-MK2 cells was evaluated using a CellTiter-Glo Luminescent Cell Viability Assay (Promega, Madison, WI), following the manufacturer’s instructions in the conditions of the focus reduction assay.

### Cell-based chemical combination assays

*In vitro* chemical combinations were performed to assess the synergism or the antagonism of suramin (**6**) in presence of a competitive inhibitor of hPIV-3 HN to block infection. Infections were performed in a focus reduction assay format, in quadruplicates in 48-well plates with ~30 focus-forming units of hPIV-3 per well, and the compounds were added post-virus adsorption to mimic post-exposure treatment. The experiments were done following the method of Chou *et al*.^36^, using constant, equipotent ratios of compounds according to their respective IC_50_ *in vitro*: 0.25-, 0.5-, 1-, 2-, and 4-fold of IC_50_. The synergism, antagonism, or additive effect of each of the combinations was assessed by calculating the combination index value (CI). According to Chou *et al*.^36^,^52^:


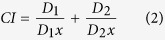


where D_1_ and D_2_ are the doses of compound 1 and 2 respectively responsible for an effect *x* in combination, while D_1_*x* and D_2_*x* are the doses of compound 1 and 2 respectively responsible for the same effect individually. If CI < 1 the compounds have a synergistic effect, if CI > 1 they are antagonistic, and if CI = 1 additive effect is observed. A normalised isobologram was created by plotting the normalised concentrations 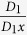 of compound 1 and 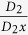 of compound 2 on the y- and x-axis respectively; where the denominators represent the respective doses of compound 1 and compound 2 alone reducing viral load by *x*%, and the numerators represent the respective doses of compound 1 and compound 2 reducing viral load by *x*% in combination. The normalised concentrations in Equation (1) were calculated given that


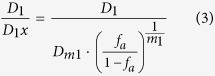


where *D*_*m1*_ is the IC_50_ of compound 1 *in vitro, f*_*a*_ is the fraction affected (or (% effect) ÷100), and *m*_*1*_ is the slope of linear regressions from median effect plots using the function


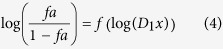


The CI-Effect graph representing the CI as a function of the associated antiviral effect was also plotted, as well as the log(DRI)-effect plot representing the log of the dose reduction index (DRI) as a function of the associated antiviral effect. The DRI is the ratio of the concentration of a compound resulting in an effect *x* alone (D_1_*x*), to the concentration of the same compound resulting in an effect *x* in combination (D_1_):


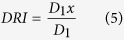


### Competition STD-NMR experiments

Purified hPIV-3 was firstly inactivated by exposure to UV light for 10 min. It was then loaded onto a 0.5 mL 30 kD cut-off concentration column (Millipore, Billerica, MA) and the virus purification buffer was exchanged at least 4 times with deuterated NaOAc 50 mM, CaCl_2_ 5 mM, pD 4.6, following the manufacturer’s instructions. For the single-compound STD-NMR, the virus was resuspended into exchange buffer to a final volume of 200 μL and suramin (**7**) or the competitive neuraminidase inhibitor zanamivir (**3**) dissolved in D_2_O was added at a final concentration of 5 mM. A control STD-NMR spectrum was acquired for each compound, in absence of virus. For competition STD-NMR experiments, the sample was reprocessed after receiving an equimolar concentration of the other inhibitor. The 1D proton (^1^H) NMR and STD-NMR spectra were acquired at 600 MHz and 283 K, as described previously^19^. For STD-NMR, the on-resonance frequency was set to −1.00 ppm and the off-resonance frequency to 300 ppm. The virus was saturated with 60 Gaussian soft pulses of 50 ms, each resulting in a total saturation time of 3 s. The residual water signal was reduced by applying the WATERGATE sequence.

### Molecular docking simulations

Simulations were carried out using the software Autodock Vina^53^. The *apo* and zanamivir (**3**)-bound structures of HN were taken from the Protein Data Bank, accession numbers 1V3B and 1V3E, respectively. Water molecules were removed, as well as B-chains to keep only one monomer. The half-suramin molecule (suramin^h,^ (**8**)), truncated after the urea moiety (NHCONH_2_-term), and suramin (**7**) were optimised through the PRODRG2 server^54^. Zanamivir (**3**) was extracted directly from the crystal structure 1V3E. Blind docking simulations were performed on the *apo* and zanamivir (**3**)-bound structures, in a 30 × 30 × 30 Å grid box (27,000 Å^3^). A total of 36 grids were designed to overlap by 15 Å in all three x, y and z directions, to reconstitute a global search space of 60 × 60 × 75 Å (270,000 Å^3^) containing an entire HN monomer. The refined dockings of suramin (**7**) and suramin^h^ (**8**) were performed in a 40 × 40 × 40 Å grid box (27,000 Å^3^) positioned in the vicinity of the HN active site, using the *apo* or the zanamivir-bound structures. Simulations were run on the Griffith University High Performance Computing Cluster “Gowonda” with exhaustiveness parameters of 16 and 512 for the blind and refined dockings, respectively. Up to 20 conformations were generated for each simulation.

## Additional Information

**How to cite this article**: Bailly, B. *et al*. A dual drug regimen synergistically blocks human parainfluenza virus infection. *Sci. Rep.* **6**, 24138; doi: 10.1038/srep24138 (2016).

## Figures and Tables

**Table 1:** Comparison of the anti-hPIV-3 haemagglutinin and neuraminidase activities of suramin (**7**) and compound **5**.

Compound	IC_50_ (μM)	
	HA	NA
Suramin (**7**)	200	13.5
Compound **5**	2.7^a^	1.5^a^

HA: haemagglutinin activity.

NA: neuraminidase activity.

^a^As reported in Guillon *et al*.^19^.

**Table 2:** Comparison of the antiviral effect of suramin (**7**) and zanamivir (**3**) or compound **5** alone or in combination.

Fold-IC_50_	Antiviral effect (%)^a^					
	Suramin (7)—Zanamivir (3)			Suramin (7)—Compound 5		
	Suramin (7)	Zanamivir (3)	Combination	Suramin (7)	5	Combination
0.25	4.88	17.17	33.22	14.47	22.79	37.60
0.5	27.91	21.27	55.03	17.97	42.13	58.00
1	46.44	46.38	89.17	55.04	54.91	95.85
2	85.54	67.31	99.76	84.56	89.87	99.89
4	99.64	95.87	99.97	99.78	99.34	99.89

^a^The antiviral effect was determined by measurement of foci size.
